# Citizens’ perspectives on relocating care: a scoping review

**DOI:** 10.1186/s12913-024-10671-3

**Published:** 2024-02-14

**Authors:** L. J. Damen, L. H. D. Van Tuyl, J. C. Korevaar, B. J. Knottnerus, J. D. De Jong

**Affiliations:** 1 Nivel, Netherlands Institute for Health Services Research, Utrecht, the Netherlands; 2 CAPHRI, Maastricht University, PO Box 616, 6200 MD Maastricht Maastricht, the Netherlands; 3 The Hague University of Applied Sciences, The Hague, the Netherlands

**Keywords:** Relocating care, Citizens’ perspectives, Primary care, Health policy

## Abstract

**Background:**

Healthcare systems around the world are facing large challenges. There are increasing demands and costs while at the same time a diminishing health workforce. Without reform, healthcare systems are unsustainable. Relocating care, for example, from hospitals to sites closer to patients’ homes, is expected to make a key contribution to keeping healthcare sustainable. Given the significant impact of this initiative on citizens, we conducted a scoping review to provide insight into the factors that influence citizens’ attitudes towards relocating care.

**Method:**

A scoping review was conducted. The search was performed in the following databases: Pubmed, Embase, Cinahl, and Scopus. Articles had to include relocating healthcare and citizens’ perspectives on this topic and the articles had to be about a European country with a strong primary care system. After applying the inclusion and exclusion criteria, 70 articles remained.

**Results:**

Factors positively influencing citizens’ attitudes towards relocating care included: convenience, familiarity, accessibility, patients having more control over their disease, and privacy. Factors influencing negative attitudes included: concerns about the quality of care, familiarity, the lack of physical examination, contact with others, convenience, and privacy. Furthermore, in general, most citizens preferred to relocate care in the studies we found, especially from the hospital to care provided at home.

**Conclusion:**

Several factors influencing the attitude of citizens towards relocating care were found. These factors are very important when determining citizens’ preferences for the location of their healthcare. The majority of studies in this review reported that citizens are in favour of relocating care. In general citizens’ perspectives on relocating care are very often missing in articles. It was significant that very few studies on relocation from the hospital to the general practitioner were identified.

**Supplementary Information:**

The online version contains supplementary material available at 10.1186/s12913-024-10671-3.

## Introduction

Demand for healthcare is increasing across the world due to a number of developments including populations ageing, technical advances in medical care, and rising incomes [1–3]. With an increase in demand, costs will also rise, while at the same time a diminishing health workforce. [1–5]. Consequently, reforms within the healthcare system will be necessary in order to control increasing healthcare costs and staff shortages [1–3]. It is assumed that reforming healthcare systems with a view to making better use of resources will make a key contribution to keeping healthcare sustainable. Estimates suggest that one fifth of health spending could be channelled towards better use, thus improving healthcare efficiency [6]. Increased efficiency could be accomplished in several ways. These may include: reducing the number of patients who receive low-value or unnecessary care; providing the same care with fewer resources, for instance by providing care in more cost-effective settings rather than in hospitals; or by reducing administrative processes that add no value [6]. This article focuses on providing care with fewer resources by relocating it to more cost-effective settings. This, in the first instance, would mean from secondary care to primary care. The thought behind this is that general practitioners (GPs) can generally provide care at less expense than hospitals for certain procedures that do not need hospital staff or environment [6]. These may include minor interventions, such as the placement of an intra-uterine device (IUD), or follow-up care, such as yearly blood- and ultrasounds, for patients who have been treated for cancer[6–9]. Relocating care to control costs could also include relocating care from secondary to homecare, self-care or eHealth [10]. Delivering care digitally can prevent a patient from having to go to the hospital. For example, an app could be used to monitor a patient receiving oxygen at home. Care commonly provided by the GP could also be relocated, to self-care, eHealth or to other healthcare providers (HCPs), like a physiotherapist or dietitian. This could result in more time for the GP to take on other secondary or primary care tasks.

It is important for relocating care to succeed, to get insights into the perspectives and needs of healthcare providers and citizens. Although involving citizens is a very important aspect of policy-making processes, it is an often overlooked form of evidence according to the World Health Organization (WHO) [11]. Citizen engagement will strengthen societal trust, will lead to more effective public policies and will lead to an improved quality of care. Furthermore, citizen engagement is essential because healthcare systems are transitioning towards a patient-centered approach, where citizens' perspectives on quality are inherently meaningful and should be a primary focus within healthcare systems [12].Extensive research has already been undertaken regarding the perspective of healthcare providers [9, 13–16], the quality and outcomes of care [17–20] and the cost perspectives [10, 17, 18, 20, 21], but not regarding the citizens' perspective on relocating care. To our knowledge, a review about citizens’ perspectives on relocating care does not exist yet. We have, therefore, conducted a scoping review with the goal of describing the findings and range of research concerning citizens’ perspectives on relocating care in more detail. A strong primary care system is required to make relocating care possible [6]. We, therefore, searched for studies that were undertaken in countries in Europe with a strong primary care [22]. Table 1 describes the characteristics of countries with strong primary care. The research questions answered in this review are: (1) Which factors influence citizens’ attitudes towards relocating care? (2) What are citizens’ preferences towards the location of care?

**Table 1:** Countries with strong primary care

Countries with strong overall primary care tend to share the following similarities [22]:
1. GPs have a central role in primary care, as they perform a gatekeeper function. They are the main point of entry to the rest of the healthcare system. GPs take on a medical advocacy role for individual patients. They monitor the health of the patients and they coordinate patient care
2. Countries with strong primary care have formally committed themselves to universal access to primary care. All these countries tend to lower the primary care co-payments, in particular for GP visits, as much as possible
Countries with a strong primary care in Europe are: the Netherlands, the United Kingdom, Belgium, Spain, Portugal, Finland, Estonia, Lithuania, Denmark, and Slovenia [22]

## Method

The aim of this review is to understand citizens’ attitudes and preferences towards relocating care. As this topic is quite broad and may be studied using many different study designs, and considering that we are not aware of any prior synthesis on this topic, a scoping review rather than a systematic review was conducted. This scoping review was carried out on the basis of the guideline by Arksey and O’Malley [23]. The review includes the following key phases: 1) identifying the research question; 2) identifying relevant studies; 3) study selection; 4) charting the data, and; 5) collating, summarising, and reporting the results.

### The search strategy and selection of literature

An initial broad search of the literature was undertaken by the first author in order to identify relevant articles that could be used for designing a search strategy. During this search, 18 key articles were identified, which included citizens, preference, and relocating care, these three terms formed the basis of our search strategy. A qualified medical information specialist was consulted in order to design and execute a sensitive search strategy. The medical information specialist also advised on which databases were most likely to contain the type of studies we were seeking and thus constituted an initial search strategy. This was refined several times after consultation. The final version was first used on the Pubmed database and then converted for each of these subsequent databases, Embase, Cinahl, and Scopus. The final search strategy, shown in Appendix A, was able to find 16 out of the 18 key articles identified. In total, it identified 19.587 articles. Duplicate references were removed, leaving 11.080 unique references. The most recent search was executed on 5 July 2022.

The selection process was performed by all authors. First, inclusion and exclusion criteria were developed. There were several inclusion criteria for this scoping review. The topic of the articles had to be citizens’ perspectives on relocating care. Only articles related to European countries with strong primary care systems were included, as a strong primary care system is required to make relocating care possible [6]. These countries were: the Netherlands, the United Kingdom, Belgium, Spain, Portugal, Finland, Estonia, Lithuania, Denmark, and Slovenia [22]. Only articles written in English, Dutch, or German were included as these were languages sufficiently mastered by the authors. In addition, all study designs were included. An overview of inclusion and exclusion criteria are shown in Table 2. In order to calibrate the inclusion process, the researchers independently applied the inclusion and exclusion criteria to a selection of three hundred articles. The task was to include, or exclude, articles based on the title alone. The results were discussed by the researchers to see if there was a maximum margin of disagreement up to 10%. This percentage was agreed in advance by the researchers. During this process, the inclusion and exclusion criteria were further refined (See Table 2). As disagreement remained, a second round of calibration was performed on 50 articles, including both titles and abstracts. The disagreement rate was now only 4% and therefore all the remaining articles were distributed among the reviewers to be scored, based on the title and abstract. After screening on the title and abstract, 167 references remained and two key articles that were not found with the search were added. These articles were distributed among the researchers once more in order to read the full text. While reading the full texts, another three relevant articles were identified through the references. These were then added too. This resulted in a total of 172 full text articles. Results from included articles were charted in a spreadsheet, which was tested by the researchers before using it. When one of the reviewers had doubts about an article, it was read by a second reviewer and the outcomes were discussed until the two researchers came to an agreement.

**Table 2:** Inclusion and exclusion criteria

Inclusion criteria	• The article includes relocating healthcare and citizens’ perspectives on this topic • The article is about a country with a strong primary care health system in Europe (Belgium, Denmark, Estonia, Finland, Lithuania, the Netherlands, Portugal, Slovenia, Spain or the United Kingdom) • The article is written in English, Dutch, or German
Exclusion criteria	• The article is not about relocating healthcare and citizens’ perspectives on this topic • The studies were before 2010
Post-hoc exclusion criteria	• Protocols • Articles about support or tools but with no relocating • Articles about self-management as a support but with no relocating • Relocating care between persons within the same institution but with no geographical relocating^a^ • The outcome measure, quality of life, is not seen as citizens’ perspective • Articles about prevention

^a^Relocating care is the act of moving healthcare from one place to another and therefore does not include task substitution, which would have been included when talking about substitution

### Data extraction

A spreadsheet was created to categorise the information that contributed to answering the research questions.

The information extracted from the articles was structured according to the type of relocation, including: relocating from the hospital to the GP, to care at home, to self-care, or to eHealth, and relocating from the GP to self-care, to care at home, or to eHealth. The difference between self-care and care at home is that self-care does not involve a healthcare provider, unlike care at home. Both forms of relocating do not involve eHealth. When the article was about eHealth it was catalogued with the eHealth category. Articles that remained, of which there was only one, were placed within the category ‘other’.

The information extracted included factors that determined citizens’ attitudes towards relocating care. All of these factors were coded by highlighter and categorised. The categories were discussed within the research team. Subsequently, we made a top three of factors for each form of relocation that occurred most often.

Furthermore, we extracted information regarding preferences for healthcare location in the articles. Citizens could have a preference for either keeping care its current location, relocating care, or a combination of both, suggesting that citizens may prefer a hybrid approach where some aspects of healthcare are relocated, while others remain in their current location. Citizens could also express equal preferences for both locations. In addition, we compared the outcomes of the one-armed, the two-armed, and the hypothetical studies, to see if there were major differences, in the preferences for healthcare location, resulting from their methodological approaches. In the one-armed studies, care was relocated for all participants in the study [24]. In the two-armed studies there was one group of participants where care was relocated, but also one group who received care as usual. The outcomes of the two groups were then compared. Hypothetical studies, presented scenarios without actual choices. They asked citizens how they would feel if care were relocated. Two-armed studies are generally considered of higher quality than one-armed and hypothetical studies, due to the presence of both an experimental group and a control group, which increases their internal validity [25].

## Results

### Search flow

**Fig. 1:**
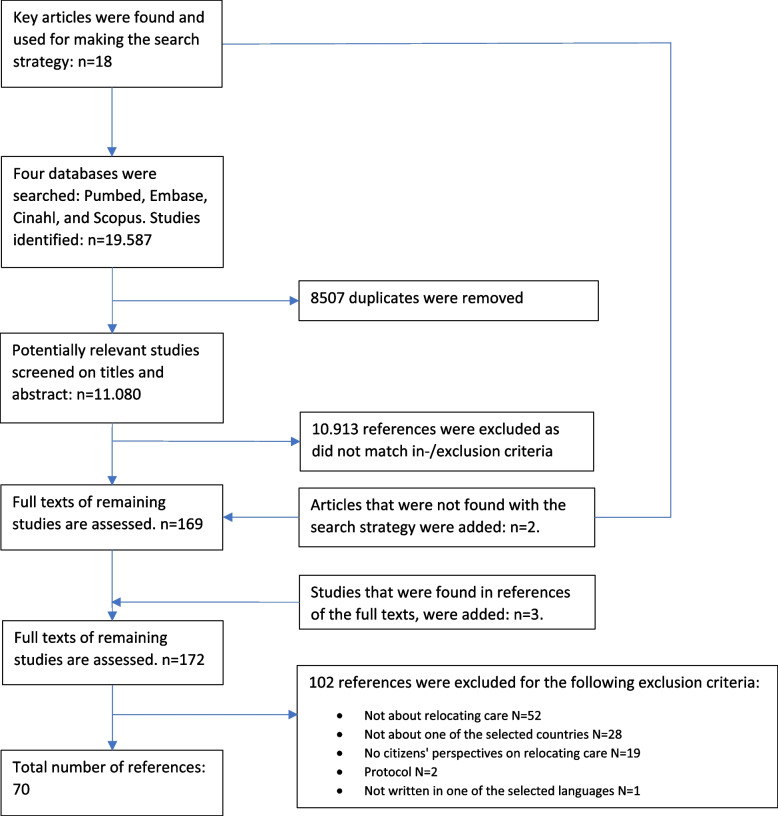
Flowchart of the review process

A total of 19,587 references were identified from the databases, of which 8,507 were duplicates, as shown in Fig. 1. At the end of the selection process, 70 full text articles were included. The characteristics of these studies are shown in Table 3.

**Table 3:** Characteristics references

First author	Year	Country	Relocation Form	Primary method	N	Target population	Arm 1/2/ hypo
Abdelmotagly	2021	UK	1	Questionnaire	100	Urology patients	1
Bager	2013	Denmark	1	Questionnaire	147	Inflammatory bowel disease patients	H
Barsom	2021	The Netherlands	1	Questionnaire	50	Colorectal cancer patients	2
Beaver	2010	England	1	Interviews	30	Breast cancer patients	1
Boydell	2021	Scotland	1, 3	Interviews	20	Women accessing an abortion service	1
Brewer	2022	England	1	Questionnaire	72	Stoma patients	1
Casey	2017	UK	1	Questionnaire	86	Prostate cancer patients	1
Damery	2021	UK	1	Interviews, questionnaire	8, 56	Liver transplant patients	2
Duncan	2019	UK	1	Questionnaire	40	(Parents of) paediatric patients	1
Hansen	2022	Denmark	1	Interviews	211	Hip osteoarthritis patients	> 2
Heeno	2021	Denmark	1	Questionnaire	280	Urology patients	1
Jones	2021	UK	1	survey	297	Rheumatology patients	1
Khan	2021	UK	1	Questionnaire	504	Possible gynaecology-oncology patients	1
Kimman	2010	The Netherlands	1	DCE	331	Breast cancer patients	H
Kjeldsted	2021	Denmark	1	Questionnaire	792	Different kinds of cancer patients	1
Knudsen	2018	Denmark	1	Interviews	15	Rheumatology patients	1
Lee	2017	UK	1	Questionnaire	32	Spinal cord injury patients	1
Lim	2022	UK	1	Questionnaire	603	Skin cancer patients	H
Lo	2021	UK	1	Questionnaire	114*	(Parents of) paediatric neurosurgery patients	1
Patel	2020	UK	1	Survey	62	Urology Patients	1
Rovira	2022	UK	1	Questionnaire	192	Possible Cancer patients (head and neck)	1
Singh	2020	UK	1	Survey	200^a^	(Parents of) paediatric patients	1
Stavrou	2021	Scotland	1	Survey	201	Neurological patients	1
Trace	2020	UK	1	Interviews	18^a^	(Families of) children using a regional paediatric nephrology service	1
Tyler	2021	England	1	Survey	2998	Citizens	1
Van Erkel	2022	The Netherlands	1	Interviews	82	Dermatology & oncology patients	1
Watters	2021	UK	1	Survey	75	Head and neck cancer patients	1
Williamson	2015	UK	1	Interviews	21	Colorectal cancer patients	1
Aicken	2016	England	2	Interviews	25	Young people (16–24) who had sex	H
Baraitser	2011	England	2	Interviews	24	Users of sexual healthcare	2
Boons	2019	The Netherlands	2	Questionnaire	61	Patients with chronic myeloid leukaemia	1
Bundgaard	2021	Denmark	2	Questionnaire	3709	Citizens above 18	1
Cameron	2010	UK	2	Questionnaire	99	Pregnant women (≤ 8 weeks gestation)	1
Den Oudendammer	2019	The Netherlands	2	Interviews, 2 focus groups	3, 26	Citizens/Users of all sorts of self-tests	1
Grogan	2017	UK	2	Questionnaire	178	Patients who are on long-term warfarin	1
Haroon	2020	UK	2	Questionnaire	44	Chronic kidney disease patients	H
Hope	2013	UK	2	Case study	1	Kidney disease patient requiring dialysis	1
Hoyos	2021	Denmark	2	Questionnaire	3725	Men Who Have Sex with Men	1
Tompson	2019	UK	2	questionnaires	140	Patients aged 40–85 years presenting with a single office systolic blood pressure between 130 and 179 mmHg	2
Tonna	2019	Scotland	2	Interviews	20	Intravenous antibiotic patients	1
Veerus	2022	Estonia	2	Questionnaire	12000	Women born between 1958–1983	> 2
Witzel	2020	UK	2	Interviews	37	Cisgender men who have sex with men	> 2
Bendien	2022	The Netherlands	3	Survey	24	Eosinophilic asthma patients	1
Corrie	2013	England	3, 4	Questionnaire, interviews	57, 11	Cancer patients	> 2
Dismore	2019	UK	3	Interviews	44	Pulmonary disease exacerbation patients	2
Goossens	2014	The Netherlands	3	DCE	107	Obstructive pulmonary disease patients	H
Hansson	2012	Denmark	3	Interviews	27^a^	(Parents of) children with cancer	1
Hansson	2013	Denmark	3	Questionnaire	185^a^	(Parents of) children with cancer	> 2
Jepsen	2016	Denmark	3	Interviews	26	Acute leukaemia patients	1
Lohr	2010	UK	3	Survey	162	Women planning an abortion	1
Rosted	2021	Denmark	3	Questionnaire	102	Citizens/Adults	2
Schiff	2022	UK	3	Questionnaire	16^a^	COVID-19 patients and close relatives	1
Uitdehaag	2014	The Netherlands	3	Questionnaire	66	Gastrointestinal cancer patients	2
Utens	2013	The Netherlands	3	Questionnaire	139	COPD patients	2
Van Ramshorst	2022	The Netherlands	3	Interviews	34	Heart failure patients	1
Baena-Cañada	2013	Spain	4	Questionnaire	98	Breast cancer patients	2
Milosevic	2021	UK	4	Interviews	25	Men with lower Urinary tract Symptoms	1
Pollard	2014	UK	4	Questionnaire	36	Cardiology patients at the GP	1
Van Bodegom-Vos	2013	The Netherlands	4	Questionnaire	694	Members of Dutch Insurance Panel	1
Van Hoof	2016	The Netherlands	4	Questionnaire	1325	Citizens	2
Wildeboer	2018	The Netherlands	4	Interviews	15	Chronic heart failure patients	H
Cottrell	2012	UK	5	Questionnaire, focus groups	124	Hypertension patients	1
Fletcher	2019	UK	5	DCE	167	Adults with hypertension (self-reported)	H
McAteer	2015	UK	5	DCE	851	Citizens	H
Scott	2020	UK	5	Interviews	6	Citizens	1
Cook	2014	England	6	9 focus groups	81	Citizens	2
Fitzsimmons	2016	England	6	Interviews, questionnaire	9, 17	COPD patients after a exacerbation	2
Heath	2015	UK	6	Interviews	27^a^	(Parents of) paediatric patients	> 2
King	2016	UK	6	Questionnaire	52	Haematuria patients	1
Mitchell	2013	UK	6	Interviews	20	Patients receiving chemotherapy	2

*H* a hypothetical study where there is no real choice, *DCE* discrete choice experiment

Forms of relocating: **1)** Hospital → eHealth; **2)** Hospital/clinic → self-care; **3)** Hospital/clinic → care at home; **4)** Hospital → GP; **5)** GP → self-care; **6)** Other

When it is indicated that a study took place in the UK, it means that it was not further specified in which country exactly or the that study took place in multiple countries within the UK

When mixed methods are involved, the N of both methods is shown in the order as the methods are named under primary method

^a^Participants of this study were children and their parents and in some cases siblings or patients and their relatives. The N shown includes both children and parents and/or siblings, or patients and relatives

The majority of studies of citizens’ perspectives on relocating care took place in the UK (*N* = 44), followed by the Netherlands (*N* = 13), and Denmark (*N* = 11). One study is from Spain and one from Estonia. Most studies are one-armed (*N* = 42), followed by two-armed (*n* = 19), and nine studies were hypothetical. While eight studies are from 2013, most studies were published quite recently in 2019 (*N* = 7), 2020 (*N* = 6), 2021 (*N* = 16), and 2022 (*N* = 9). Relocating care from the hospital to eHealth is the form of relocating that is most often examined within the studies identified (*N* = 28) [26–53]. This is followed by relocating from the hospital to self-care (*N* = 15) [54–67] and care at home (*N* = 13) [30, 68–80]. Forms of relocating care that are not frequently studied include relocating from the hospital to the GP (*N* = 7) [16, 69, 81–85] and from the GP to self-care (*N* = 4) [86–89]. Five more forms of relocating are listed under the heading “other”. These include: relocating from the hospital to a community-based clinic [90]; from outpatient visits to a one-stop clinic [91]; nurse home visits that were replaced by eHealth [92]; hospital care relocated to a mobile chemotherapy unit [93]; and, care relocated from the GP to eHealth [94]. Most studies are about the relocation of care for oncology patients (*N* = 19), followed by citizens in general (*N* = 10), and cardiology patients (*N* = 8).

### Which factors influence citizens’ attitudes towards relocating care?

#### Convenience

The most frequently cited factors influencing citizens’ attitudes towards relocating care are shown in Table 4. Convenience was most often reported, from the citizens’ perspective, as an advantage of relocating care. This was true for all forms of relocation [27–30, 32–34, 38, 41, 42, 45, 47, 49, 52–54, 58–60, 65–67, 69, 70, 73, 78, 82, 84–86, 88, 90, 93, 94]. Citizens think of relocating as convenient because in most cases it saves travel time [26, 29, 53]. It saves costs [26, 69]. It avoids stress due to factors such as transport problems, busy traffic, travelling while you are sick, or long sojourns in waiting rooms [26, 53, 73, 93]. When relocating to self-care it was very often mentioned that it is an advantage to have more flexibility [30, 86]. Citizens can do a self-test whenever and wherever they want, without having to consider opening hours, for example [59, 66, 67]. Convenience was also mentioned as a reason for *not* wanting to relocate care. This factor was especially mentioned when relocating from the hospital or GP to self-care [59, 60, 86]. With regard to home dialysis, some citizens said that they did not have the space at home to do this. It was, therefore, not convenient [60]. In addition, for citizens living close to the hospital, self-care was sometimes more expensive and did not save time [59, 86].

**Table 4:** Most named factors influencing citizens’ preferences for relocating care

	**Factors for having a *positive* attitude towards relocating care**		**Factors for having a *negative* attitude towards relocating care**	
1	Convenience	^1, 2, 3, 4, 5, 6^	Quality of care	^1, 2, 3, 4, 5, 6^
2	Familiarity	^1, 3, 4, 5, 6^	Familiarity	^1, 2, 4, 5, 6^
3	Accessibility	^4, 5, 6^	No physical examination	^1, 5, 6^
4	Patients have more control over their disease	^1, 2, 3, 5^	Contact with others	^3^
5	Privacy	^2, 3^	Convenience	^5^
6			Privacy	^3^

The third and fifth columns show the forms of relocating care where the factor mentioned, occurred in the top three most mentioned factors

Forms of relocating: **1)** Hospital → eHealth; **2)** Hospital/clinic → self-care; **3)** Hospital/clinic → care at home; **4)** Hospital → GP; **5)** GP → self-care; **6)** Other

#### Familiarity

Familiarity was another factor which was reported as important to citizens regarding their attitude towards relocating care [29–33, 58, 61, 67–70, 73, 74, 77, 83–86, 90, 94]. Some citizens feel more familiar with their GP than with a hospital specialist and would, therefore, want to relocate care [83, 84]. Other citizens experience a sense of familiarity due to the environment in which care is provided. When receiving care at home, citizens feel more familiar, because they are in their own environment with their own support system [29, 30, 50, 58, 70, 77]. In addition, when receiving care at home, the HCP enters the personal space of the patient. This, according to some of the patients, provided a better and more personal connection with the HCP. As shown in Table 4, familiarity is also named as a reason *not* to want to relocate. While some citizens said that they had a better relationship with their GP, others said they were more familiar with the specialist so they would rather go there [85]. Some citizens thought that personal contact was reduced when using eHealth. They felt that it was more distant [31, 33, 36, 47, 51]. In addition, during telephone consultations, citizens did not feel a sense of familiarity if they had never seen the HCP before and therefore could not picture the face belonging to the voice. [29]. With regard to self-care, some citizens did not feel a sense of familiarity because this care is usually performed alone, while they preferred to have the support of a HCP [60, 63].

#### Accessibility

The third most frequently mentioned factor that influenced citizens’ perceptions of relocating care was “accessibility”. Citizens were more willing to relocate care when waiting times became shorter and so the accessibility became better [28–30, 45, 49, 54, 58, 82–84, 88, 90, 91, 93]. For example when relocating from the hospital to the GP [82–84]. Regarding self-tests, citizens mentioned that they had very rapid access. They can pick up the test and then apply it directly, without having to make an appointment with a HCP, who is often not immediately available [30, 54, 55, 58]. In addition, with a self-test you often get the results without delay [55, 59]. With regard to eHealth, citizens said that access to the HCP improved because they could contact them easily when they had questions [28, 49].

#### Patients have more control

Another advantage of relocating care, mentioned by citizens, is being more in control, especially when relocating care from the hospital to eHealth, self-care, or to care at home [30, 54, 58, 60, 70, 73]. The sense of increased control can stem from two primary factors. Firstly, patients become more actively engaged in their healthcare, leading to a better understanding of their diagnoses and consequently, greater control over their condition [38, 49, 53, 59, 86]. Secondly, citizens felt more involved in the process of decision making regarding their healthcare, affording them the ability to influence what happens and when [49, 50, 59, 74]. This gives them the feeling of having more control over their lives.

#### Privacy

The last factor named as an advantage, but also as a disadvantage of relocating care, is ‘privacy’. Citizens who saw it as an advantage mentioned that there is more privacy at home using eHealth or self-care than there is in a hospital [53–55, 58, 60, 66, 69, 70, 74]. With regard to self-care there are a lot of articles about using self-tests to check for sexually transmitted infections or about administering drugs oneself at home in order to induce an abortion. Citizens indicated that having such tests carried out at a clinic may cause a lot of embarrassment [54]. You may run into acquaintances for example [67]. Self-care, on the other hand, is more anonymous and thus offers more privacy [55]. However, privacy is also named as an disadvantage by citizens. Regarding eHealth, some citizens are concerned about whether the privacy of their data can be guaranteed [33]. In addition, some citizens said that it was hard to find a private space in their house during the covid-19 crisis [30]. Furthermore, when care is being given at home, some citizens do not like the fact that other family members may witness them being treated [69] or that caregivers are having to enter their home, thus violating their privacy [70].

#### Quality of care

The most frequently mentioned factor for having a negative attitude towards relocating care is that citizens have concerns about the quality of care when care is being relocated, due to less expertise of the HCP or insufficient quality of the instrument or self-test, which will be involved in the new location [28, 32–34, 36, 47, 51, 54, 55, 59, 60, 63, 65, 67, 69, 70, 73, 77, 82, 85–87, 90, 94]. Regarding relocating care to eHealth or self-care a lack of trust in eHealth technology [33, 34, 36, 47], or a particular self-care device, [54, 55, 59, 60, 63, 65, 67] was reported very often. Citizens fear technical problems or that important factors might be overlooked. Neither do some citizens feel that they have the right skills for using the new eHealth technology [36] or performing self-care in the right way [54, 60, 65, 67]. Regarding care at home, citizens were concerned with the absence of constant surveillance and a diminished contact with the doctor. Moreover, citizens felt that the hospital is better equipped [77]. With regard to relocating from the hospital to the GP, some citizens thought that the specialist had more expertise which was a reason for them not wanting to relocate [82, 85]. 

#### No physical examination

Another factor for not wanting to relocate care is where it results in an absence of physical examination. This reason was named many times when relocating care from the hospital to eHealth [27, 29, 31, 34, 47, 51, 52] and relocating from the GP to self-care [86, 89]. With regard to eHealth, some citizens say that they found it difficult because they are not able to demonstrate physical symptoms and they find it hard to describe problems without seeing the HCP [31, 33].

#### Contact with others

The last factor, frequently mentioned as a disadvantage of relocating care, is less contact with their peers. This aspect was most mentioned regarding relocating from the hospital to care at home [69, 70, 73]. Some citizens enjoyed going to the hospital because of the social interaction with other citizens. They were afraid of social isolation [60].

### What are citizens’ preferences regarding the location of care?

A total of 49 articles investigated citizens’ preferences regarding the location of healthcare. Their location preferences for each form of relocating care will be discussed below and are shown in Table 5.

**Table 5:** Preferences for relocating care

	Hospital –eHealth	Hospital –self-care	Hospital – care at home	Hospital – GP	GP – self-care
Total (N)	23	8	10	5	2
Preference relocating	10	5	8	1	1
Preference not relocating	6	3	0	2	1
Combination	4				
Preferences equal	3		2	1	

Within the articles about relocating from the hospital to eHealth, 23 articles out of 28 provided the preferences of respondents towards the location of care. In ten articles there was a preference for eHealth [28, 32–34, 42, 44–46, 50, 53] and in six articles a preference for the hospital [26, 31, 36, 39, 43, 48]. In four articles, citizens expressed a wish for a combination of eHealth and face to face contact [37, 47, 49, 52]. In the remaining articles (*N* = 3), the preference was equal for the hospital and for eHealth [35, 41, 51].

Eight out of 15 articles about relocating from the hospital to self-care investigated citizens preferences for the location of care. In five articles citizens showed a preference for self-care [56, 57, 61, 64, 66] and in three articles for the hospital [55, 60, 65].

With regard to articles about relocating from the hospital to care at home, ten out of 13 articles investigated a preference for healthcare location. In eight articles, the participants had a preference for care at home [68, 69, 72, 74, 75, 78–80]. In two articles, preferences for care at home and the hospital were equal [71, 76]. There were no articles with a preference for the hospital.

Regarding relocating from the hospital to the GP, there were five out of seven articles investigating citizens preferences regarding healthcare location. In two articles, participants preferred the hospital over the GP [81, 85]. In one they preferred the GP [84], and in the other, preferences were equal [16]. In the fifth study citizens could choose between three locations: the hospital, the GP, or care at Home. Here they preferred care at home followed by care at the general practice [69].

Two out of four articles about relocating from the GP to self-care investigated a preference for a healthcare location. In one article, citizens preferred self-care [86], and in the other, they preferred the GP [89].

Within the category “other”, there were two articles which investigated a preference for a healthcare location. In the article about relocating from the hospital to one-and-a-half line care, citizens preferred one-and-a-half line care [91]. The last article was about nurse home visits that were relocated to eHealth. Here, citizens preferred eHealth over the nurse visits [93].

Most articles adopted a one-armed approach. Since two-armed articles are often of higher quality, we compared the results of the one-armed, and the two-armed, articles. In total there were 19 two-armed articles of which 14 investigated a preference for healthcare location. In nine out of 14 articles citizens preferred relocating healthcare and in two articles they did not. In the other articles, preferences were equal. Of the 35 one-armed articles which investigated healthcare preferences in 18 articles, citizens gave a preference for relocating healthcare. Thus, in both cases, there is a preference for relocating care in just over half of the articles. We see here a different outcome than with the hypothetical studies (*N* = 10). Here there was *no* preference for relocating care in five out of seven articles.

## Discussion

This scoping review was conducted in order to provide insight into the factors that influence citizens attitudes towards relocating care. Seventy articles were included and most which were found were about relocating care from the hospital to eHealth. Most of these articles about eHealth were published in 2020 or later (*N* = 20). Only eight articles were published in 2019 or earlier. This is likely due to covid-19, which started in 2020 in Europe and required healthcare providers in many places to offer care online.

The first research question concerned which factors influence citizens attitudes towards relocating care. The most frequent reported factor for a positive attitude towards relocating care is “convenience”, according to citizens, followed by “familiarity”. Other factors that were in the top three of reasons for a positive attitude towards relocating care were “accessibility”, “patients have more control”, and “privacy”. The positive drivers for relocating care are almost the same for all forms of relocating. The two most mentioned factors for a negative attitude towards relocating care are, first of all, citizens having concerns about the quality of care and, secondly, citizens feel less familiar when care is being relocated. Other reasons to have a negative attitude towards relocating are “the lack of physical examination”, “contact with others”, “convenience”, and “privacy”.

The second research question concerned citizens’ preferences for healthcare location. In general, as far as the conditions and treatments mentioned in the articles are concerned, most citizens favoured relocating healthcare. Especially with regard to care at home, there were no articles found where citizens had a preference for the hospital instead of care at home. In addition, eHealth and self-care are also carried out from home. Citizens thus prefer receiving care at home.

Not all articles investigated preferences for the location of healthcare, and of those which did, most were one-armed. However, there were no major differences found when comparing the outcomes of the one-armed and two-armed studies. This contrasted with the hypothetical studies, where citizens did not prefer relocating care in the majority of cases. This may be due to the fact that citizens are familiar with the current situation and do not know, or find it difficult to imagine, what a new situation will look like. Citizens may not want to relocate because familiarity is an important aspect of healthcare, as described earlier.

The articles found included a wide variety of conditions and phases of treatment. We would have preferred to distinguish between different conditions and treatment phases, as these aspects may determine the preference for healthcare location. For example, it might be the case that citizens would like to relocate follow-up cancer care to care at home, while keeping the treatment itself in the hospital. However, the large variation in conditions and phases of treatment resulted in a small N per condition or phase of treatment and this hampered further in-depth analysis.

Relocating care often involves not only the location changing, but also other aspects. For instance, the care provider may change too, for example a telephone consultation with a nurse instead of a face to face appointment with the specialist in the hospital [32, 53]. And in some cases, the purpose of treatment changed, for example, a telephone consultation that was meant for providing information and supporting patients, while a face to face consultation was more focused on looking for signs of recurrent disease [29]. All of these factors together determine the preference for healthcare location. So it is not only the location on which citizens base their preference. It is, therefore, important to take all aspects into account, not only the geography when investigating the preferences for healthcare location.

### Strengths and limitations

A strength of this scoping review is that it has a broad search strategy developed together with a medical information specialist. This resulted in over 11.000 references that were all assessed. However, the search strategy may not have been broad enough, as some articles were missed, including two of the 18 key articles. This was known beforehand and so we investigated why the two key articles were not found. One key article was not found because we did not use the word “experience” [16] while the other focused on the terms “breast cancer”, “follow-up care”, and “healthcare models” [81], which we did not use in our search strategy. The words used in these two articles were not words we saw repeated in other relevant articles. Adding any of the key words yielded about 5,800 additional results in Pubmed alone. Therefore, we chose to add the key articles manually and left these words out of the search string. All statements made in this article are based on the conditions and forms of care that recurred in the studies we found. There may be other forms of care that could be relocated that have not been discussed in this article.

Another limitation of this study is that the articles are not double reviewed because of the large number of references found. However, to calibrate the inclusion process, the researchers applied the inclusion and exclusion criteria to a selection of 350 articles. Also, it was decided to start with reviewing abstracts, instead of titles, which is the normal procedure [23].

A limitation of a scoping review is that it analyses studies that use a range of data collection and techniques. This makes it more difficult to synthesise the results of the studies [23]. A strong point of this review is that we made a comparison between one-armed and two-armed articles and that approximately the same results emerged in the articles.

### Research implications

A knowledge gap we identified is that citizens’ perspectives on relocating care received relatively little attention within the current literature. In particular, we found limited literature focusing on citizens’ perspectives regarding the relocation of care from the hospital to the GP. This gap is significant, because this is one of the forms of relocating that governments think of first in order to limit healthcare costs [6–8]. There are several studies about this subject but they do not involve the citizens’ perspective. Despite the importance of including citizens' perspectives in policy-making processes, it often remains underrepresented in the literature [11]. The World Health Organization (WHO) emphasizes that citizen engagement can enhance societal trust and lead to more effective public policies.

Another knowledge gap we identified is that insufficient research has been done on different treatment phases and conditions in healthcare with regard to citizens’ perspectives and relocating care. To fill this gap, future research should delve deeper into the relationship between the factors leading to particular attitudes towards relocating care, and preferences for location of care and different conditions and treatment phases, including diagnosis, treatment phase and aftercare.

Our study has also revealed practical implications that can inform healthcare policy and decision-making. Firstly, the factors we have identified can serve as conditions that governments can use to improve acceptance among citizens regarding healthcare location. They can be used as conditions that have to be met, and that can be used to direct citizens to a particular location. Secondly, it's evident from our findings that citizens generally prefer receiving care from home. This preference presents an opportunity for governments to invest in home-based healthcare services, potentially leading to higher citizen satisfaction and more cost-effective healthcare delivery.

## Conclusion

Positive factors influencing the attitude of citizens towards relocating care are almost the same for all forms of this development—with convenience as the most important. The most often reported factor for having a negative attitude towards relocating care are concerns about the quality of care. The factors found are very important when determining a citizens’ preference for a particular healthcare location. The majority of studies in this review reported that citizens are in favour of relocating care, especially to care at home. Several knowledge gaps were identified. Strikingly, very few studies on relocation from the hospital to the GP were identified.

### Supplementary Information

**Additional file 1: Appendix A.** Search string Pubmed.

## Data Availability

Not applicable. The studies we used are accessible to everyone. All studies used are included in the references.

## Abbreviations

GP: General practitioner
HCP: Healthcare provider
IUD: Intra-uterine device
