# Supplementary material for: Citizens’ perspectives on relocating care: a scoping review
Source: BMC Health Serv Res. 2024 Feb 14;24:202. doi: 10.1186/s12913-024-10671-3 (PMC10868012; doi:10.1186/s12913-024-10671-3)
Supplement: Supplementary file 1 — Additional file 1: Appendix A. Search string Pubmed. [file 12913_2024_10671_MOESM1_ESM.docx]

**Appendix A – Search string Pubmed**

Below is the search string used in Pubmed:

("Outpatient Clinics, Hospital"[Mesh] OR "Self Care"[Mesh] OR “outreach clinic*”[tiab] OR “outpatient clinic*”[tiab] OR “outpatients clinic*”[tiab] OR "out-patient clinic"[tiab] OR "outpatients

clinic"[tiab] OR “outpatient care”[tiab] OR "out-patient care"[tiab] OR “outpatient setting”[tiab] OR "out-patient setting*"[tiab] OR "outpatient department*"[tiab] OR "outpatients department*"[tiab] OR "out-patient department*"[tiab] OR "outpatient service*"[tiab] OR hospital at-home[tiab] OR “treatment place*”[tiab] OR “treatment setting*”[tiab] OR (substitution[tiab] AND care[tiab]) OR

“primary care plus”[tiab] OR hospital-at-home[tiab] OR selfcare[ tiab] OR selfcare[tiab]) AND ("Patient Preference"[Mesh] OR prefer*[tiab] OR priorit*[tiab] OR choic*[tiab] OR DCE[tiab] OR “discrete choice experiment*”[tiab] OR “conjoint analys*”[tiab] OR perspective*[tiab] OR expectation*[tiab] OR perceiv*[tiab] OR perception*[tiab]) AND ("Netherlands"[Mesh] OR "Belgium"[Mesh] OR "United Kingdom"[Mesh] OR "Spain"[Mesh] OR "Portugal"[Mesh] OR

"Finland"[Mesh] OR "Denmark"[Mesh] OR "Estonia"[Mesh] OR "Lithuania"[Mesh] OR "Slovenia"[Mesh] OR netherland*[tiab] OR dutch[tiab] OR holland*[tiab] OR belgium[tiab] OR

belgian*[tiab] OR “united kingdom”[tiab] OR “UK”[tiab] OR britain[tiab] OR british[tiab] OR briton*[tiab] OR england[tiab] OR english[tiab] OR (wales[tiab] NOT new-south-wales[tiab])OR

welsh*[tiab] OR Scotland[tiab] OR Scottish[tiab] OR northernireland[ tiab] OR north-Ireland[tiab] OR north-irish[tiab] OR spain[tiab] OR spanish[tiab] OR spaniard*[tiab] OR portugal[tiab] OR portug*[tiab] OR finland[tiab] OR finn*[tiab] OR estonia*[tiab] OR lithuania*[tiab] OR denmark[tiab] OR danish[tiab] OR dane*[tiab] OR sloven*[tiab] OR netherlands[ad] OR dutch[ad] OR holland*[ad] OR belgium[ad] OR belgian*[ad] OR “united kingdom”[ad] OR britain[ad] OR british[ad] OR briton*[ad] OR england[ad] OR english[ad] OR (wales[ad] NOT new-south-wales[ad]) OR welsh*[ad] OR scotland[ad] OR scottish[ad] OR northern-ireland[ad] OR north- Ireland[ad] OR north-irish[ad] OR spain[ad] OR spanish[ad] OR portugal[ad] OR portug*[ad] OR finland[ad] OR finn*[ad] OR estonia*[ad] OR lithuania*[ad] OR denmark[ad] OR danish[ad] OR dane*[ad] OR sloven*[ad] OR netherlands[pl] OR belgium[pl] OR england[pl] OR wales[pl] OR scotland[pl] OR northern-ireland[pl] OR spain[pl] OR portugal[pl] OR finland[pl] OR estonia*[pl] OR lithuania*[pl] OR denmark[pl] OR

sloven*[pl])
